# The Mid-Domain Effect Shapes a Unimodal Latitudinal Pattern in Fruiting Phenology

**DOI:** 10.3390/plants14233701

**Published:** 2025-12-04

**Authors:** Longyang Zhang, Qianhuai Xue, Yanjun Du

**Affiliations:** School of Tropical Agriculture and Forestry, Hainan University, Haikou 570228, China

**Keywords:** geometric constraints, latitudinal gradient, macroecology, macrophenology, null model, temporal niche, phenological diversity

## Abstract

The mid-domain effect (MDE) has been used to explain spatial diversity patterns and flowering phenology, but its role in fruiting phenology has received limited attention to date. This study investigates whether the MDE shapes fruiting phenology and whether its influence varies with latitude. We integrated fruiting phenology data for 12,179 plant species across 28 Chinese provinces and used a null model to simulate expected fruiting richness patterns. Our results suggest that the MDE plays a significant role in explaining fruiting phenology patterns in most provinces. Crucially, the variance explained by the MDE exhibited a significant unimodal relationship with latitude across all groups, peaking at mid-latitudes (39.6° N for all species, 37.1° N for herbaceous plants, and 36.8° N for woody plants). Unlike flowering phenology—which tends to show a simple linear increase in MDE strength with latitude—fruiting exhibited a distinct peak, highlighting different ecological pressures acting on these two reproductive stages. The MDE was the primary contributor explaining fruiting richness, providing a markedly stronger fit to the data than key climate variables like temperature and precipitation, although woody plants showed a stronger secondary response to precipitation. These findings demonstrate that geometric constraints are a key driver of fruiting phenology, deepening our understanding of temporal niches and the ecological processes shaping plant reproductive phenology.

## 1. Introduction

The seasonal timing of fruiting is a critical life-history trait that underpins plant reproductive success and ecosystem function [1,2]. Within the fruiting period, most plant species exhibit a distinct peak in fruit production. This peak creates a crucial window for regeneration that influences seedling establishment [3]. Furthermore, as a peak in resource abundance, it shapes animal foraging behavior and thus governs seed dispersal dynamics [4]. Shifts in the timing of peak fruiting can trigger cascading effects throughout ecological communities via trophic interactions. Over time, such changes can drive significant restructuring of plant community composition [1,4,5,6]. When considering environmental controls on fruiting it is useful to distinguish proximate drivers, such as temperature, precipitation, and light availability, which directly regulate fruit development, from ultimate evolutionary factors, including geometric constraints and seed-predation pressure, which shape long-term reproductive strategies [7,8,9].

The mid-domain effect (MDE) hypothesis was originally proposed to explain geographical patterns of species richness, particularly the latitudinal diversity gradient [10,11]. It posits that when species’ geographical ranges are randomly placed within a bounded domain, the overlap of these ranges is inevitably greatest in the center of the domain, creating a peak in species richness [10]. This geometric constraint model has been successfully applied to explain diversity patterns along both latitudinal and elevational gradients [11,12,13,14]. More recently, the concept of the MDE has been extended by analogy from spatial to temporal dimensions to explain phenological patterns [15]. This extension is motivated by the methodological similarity in the null models used to assess both spatial diversity gradients and phenological overlap. It is critical to note that this analytical analogy does not assume that fruiting duration distributions are necessarily random processes; rather, it provides a neutral baseline against which observed patterns can be compared. Research has shown that when species’ flowering periods are randomly distributed within a constrained seasonal window, the richness of flowering species also tends to peak near the temporal midpoint, revealing a temporal mid-domain effect [16,17]. This extension underscores the model’s utility in uncovering the role of geometric constraints in structuring ecological communities across time.

**Figure 1:**
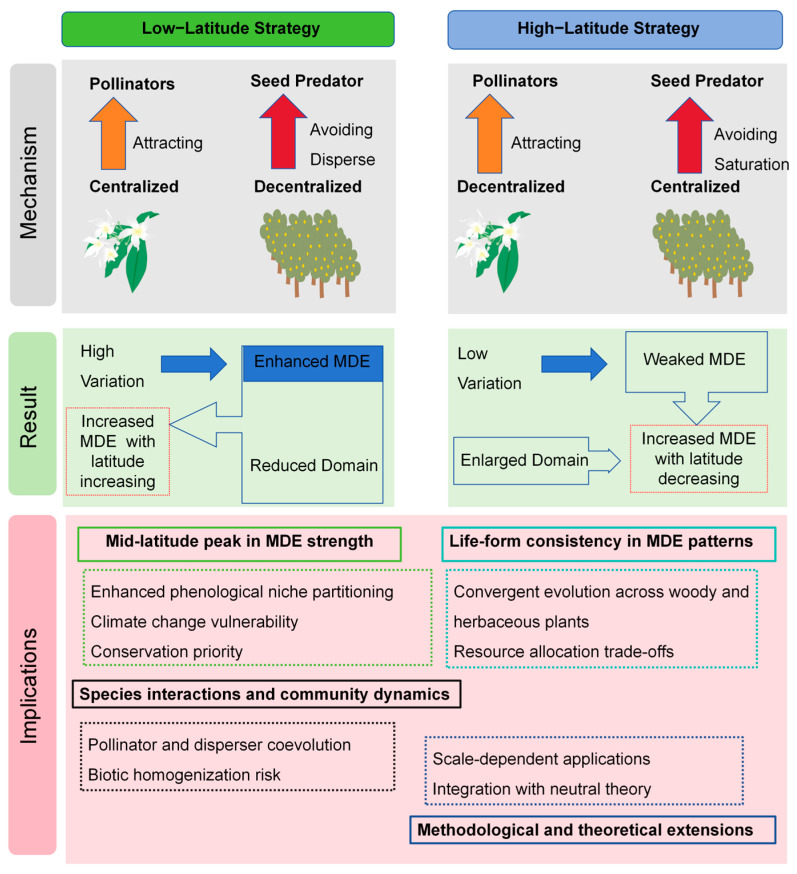
Latitudinal variation in the mid-domain effect (MDE) under selective pressures: mechanisms, results, and potential eco-evolutionary impacts. The intensity of the MDE is influenced by the size of the domain and the duration of the phenological periods. Specifically, a larger domain or a shorter phenophase duration can attenuate the MDE. Divergent survival strategies in plant’s flowering and fruiting, shaped by varying selective pressures, lead to distinct latitudinal gradients in phenophase duration. This, in turn, predisposes the evolution of divergent latitudinal patterns in MDE strength.

Previous studies have established a positive correlation between the explanatory power of the MDE in flowering phenology and latitude [16]. This pattern challenges the traditional paradigm derived from spatial niche studies [18,19,20,21] and highlights the need for a more refined conceptual perspective to understand community assembly in temporal niches, where geometric constraints may play a more fundamental role. This discrepancy suggests that theories governing spatial niches may not directly apply to temporal niches. Building upon this, we propose a theoretical framework (Figure 1) that predicts a distinct latitudinal pattern for fruiting phenology, potentially divergent from that of flowering. The underlying rationale is that flowering and fruiting are subject to different selective pressures: flowering is primarily optimized for pollinator attraction, whereas fruiting is shaped by strategies to escape or satiate seed predators. A key ecological hypothesis, which this study aims to test, is that intense seed predation may select for greater intraspecific temporal dispersion of fruiting [4,22]. This would allow plants to reduce per capita predation risk through temporal escape rather than synchrony. Global empirical studies show that seed predation intensifies fivefold from high to low latitudes [23]. Based on this empirical pattern, we hypothesize that at low latitudes, plants concentrate flowering to match specialized pollinators but disperse fruiting to mitigate high predator pressure. Conversely, at high latitudes, plants disperse flowering to maximize pollination success in a short season but concentrate fruiting to achieve predator satiation. Crucially, based on the opposing latitudinal trends, we predict a unimodal (bell-shaped) relationship between the explanatory power of the MDE and latitude. Specifically, we expect this relationship to peak at mid-latitudes (approximately 35° N–40° N), with positive curvature (increasing explanatory power) at lower latitudes and negative curvature (decreasing explanatory power) at higher latitudes. Conceptually, the two latitudinal trends—domain contraction (which strengthens geometric overlap) and phenophase shortening (which reduces overlap)—do not change at the same rate. At low latitudes, the long domain imposes weak geometric constraints regardless of species durations; at high latitudes, phenophase durations become so short that geometric constraints again have little room to operate. Only at mid-latitudes do domain size and phenophase breadth fall into a range where geometric overlap is maximized, producing the unimodal peak. This nonlinear interaction between domain size and species-level duration is the mechanistic basis for the unimodal pattern (Figure 1).

Based on this framework, the present study aims to empirically test three key hypotheses:

(1) The mid-domain effect significantly explains the pattern of peak fruiting times at a regional scale;

(2) The explanatory power of the MDE for fruiting phenology exhibits a unimodal, rather than linear, relationship with latitude, peaking in mid-latitude regions;

(3) The fruiting phenology of both herbaceous and woody plants is associated with the MDE, albeit potentially with differing sensitivities to climate variables.

To test these hypotheses, we integrated a comprehensive dataset comprising the phenological records of 12,179 herbaceous and woody plants from provincial Floras and species distribution data from the Chinese Vascular Plant Distribution Database. We employed a null model approach to generate MDE-driven predictions of monthly fruiting richness and assessed their fit with empirical observations. Furthermore, we quantified the relative contributions of the MDE and key climatic factors—namely, mean monthly minimum temperature, mean monthly precipitation, and mean monthly sunshine duration—to the observed fruiting patterns.

## 2. Results

### 2.1. Mid-Domain Effect in Peak Fruiting Phenology

**Figure 2:**
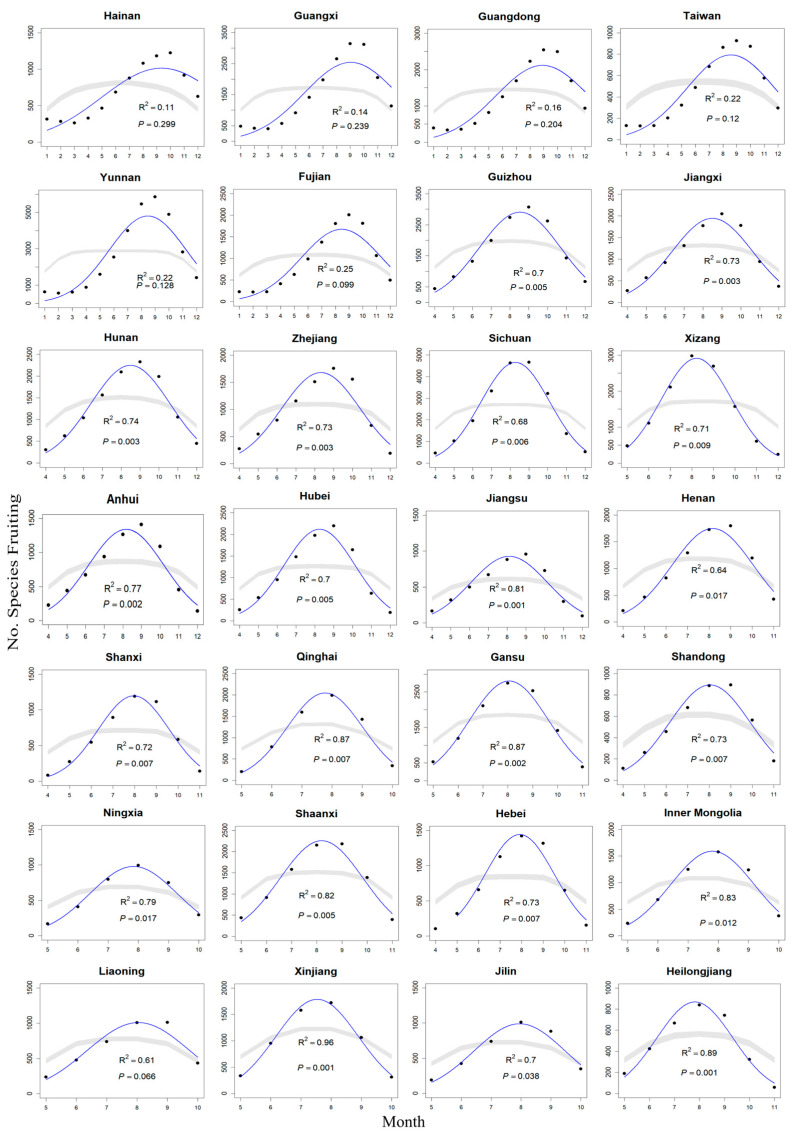
Observed (black dots) and null model-predicted fruiting diversity for 28 provinces, based on the 5th and 95th percentiles of cumulative fruiting records. The fitted values (solid lines) and the 95% confidence interval predicted by the mid-domain models (gray bands) are shown in the plots. Each panel represents one province, labeled with the province name. The R^2^ and *p*-values reflect the relationship between the observed data and the predicted values from the mid-domain model.

**Figure 3:**
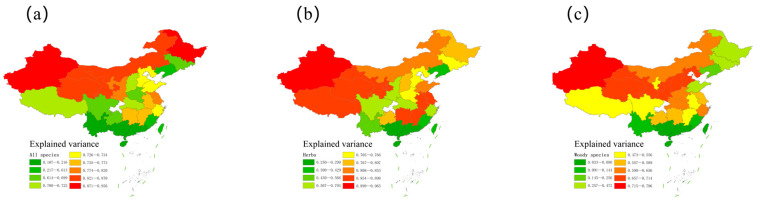
The geographic patterns of the variance explained by the mid-domain effect models in China estimated at the provincial level for (**a**) all species pooled, (**b**) herbs, and (**c**) woody species based on the 5th and 95th percentiles of cumulative fruiting records.

The null model, based on the 5th and 95th percentiles of cumulative fruiting records, explained a statistically significant fit (*p* < 0.05) of the variation in fruiting species richness across the temporal gradient in 21 out of 28 provinces for all species combined (Figure 2). This pattern was more pronounced in herbaceous species, with a significant fit between empirical and predicted richness in 24 out of 28 provinces (Table 1 and Figure 3b). Notably, the explanatory power of the MDE varied substantially, with Hainan (tropical climate) and Xinjiang (arid climate) representing the lowest and highest R^2^ values, respectively. In contrast to provinces with significant fits, the seven non-significant provinces exhibited only weak geometric structure, with R^2^ values generally below 0.30 (Table 1). These low values indicate that temporal geometric overlap is minimal in regions where fruiting spans a large portion of the year or where phenological records are more diffuse. For woody species, the model captured the observed pattern in 16 provinces (Table 1 and Figure 3c). The variance in fruiting species richness explained by the mid-domain effect model ranged from 10.7% to 95.6% for all species (mean = 63.7%, 95% CI: 53.8–73.6%), from 25.0% to 96.5% for herbaceous species (mean = 69.8%, 95% CI: 61.3–78.2%), and from 3.3% to 79.6% for woody species (mean = 47.8%, 95% CI: 38.9–56.8%) (Table 1). Distribution of MDE explanatory power across provinces showed consistent left-skewness (negative tail asymmetry indices: all species = −0.428, herbs = −0.369, woody = −0.328).

### 2.2. Latitudinal Patterns of the Mid-Domain Effect

**Figure 4:**
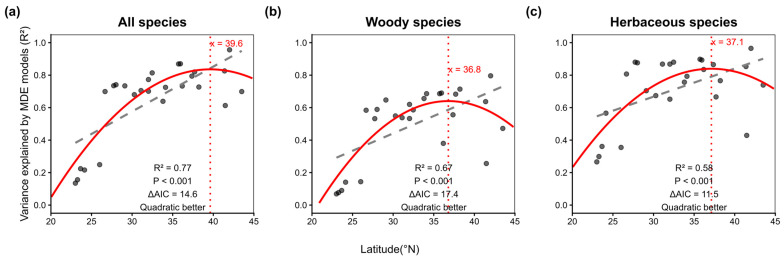
The relationship between the variance explained by the mid-domain effect models and latitude for all species (**a**), woody species (**b**) and herbaceous species (**c**), based on the 5th and 95th percentiles of cumulative fruiting records. Each circle represents a single province. The solid red curve is the fitted line from the nonlinear model. The gray dashed line is the fitted line from the linear model. The red vertical dashed line represents the peak of the quadratic regression curve. ∆AIC is the difference in AIC between two models, and a ∆AIC greater than zero indicates better fitting of the nonlinear model.

Analyses using the 5th domain definition revealed a consistent unimodal relationship between the variance explained by MDE models and latitude across all taxonomic groups. Because the quadratic models consistently showed substantially lower AIC values than the linear models (all ∆AIC > 0), we selected the quadratic form as the final model describing the latitudinal variation in MDE explanatory power. For all species combined, the quadratic peak occurred at 39.6° N, with a negative curvature coefficient (−0.002, *p* < 0.001) indicating a well-defined unimodal pattern (Figure 3 and Figure 4a; n = 28, ∆AIC = 14.6, R^2^ = 0.77). Woody (Figure 4b; n = 28, ∆AIC = 17.4, R^2^ = 0.67) and herbaceous species (Figure 4c; n = 28, ∆AIC = 11.5, R^2^ = 0.58) exhibited similar peak locations (36.8° N and 37.1° N, respectively). The quadratic curves are broadly symmetrical around the mid-latitude peak, although the two tails are not perfectly mirror images. This slight deviation from perfect symmetry simply reflects the empirical pattern shown in Figure 4.

### 2.3. Relationship Between Climatic Variables and Fruiting Diversity

Linear mixed-effects models revealed that the observed monthly number of fruiting species was significantly correlated with both the mid-domain effect (MDE) predictions and temperature variables across all species, a pattern that held for herbaceous plants specifically (Table 2; *p* < 0.01). The semi-standardized coefficient for MDE (Table S2; all βss > 0.70) was substantially larger than those of the climate variables. In contrast, woody plants showed significant correlations not only with MDE predictions and temperature but also with monthly precipitation (Table 2; *p* < 0.01). For herbaceous plants, the MDE model demonstrated strong predictive power, explaining 92.96% of the observed variation in fruiting species richness. Tmin accounted for 4.41% of the variation, while MMP and Sunshine each explained less than 5%, indicating negligible independent contributions once the geometric constraint is considered. Similarly, the MDE model for woody plants also showed strong predictive ability, explaining 72.85% of the variation. Tmin and MMP were the second and third most important predictors, explaining 15.50% and 6.71% of the variation, respectively.

## 3. Discussion

### 3.1. Interpretation of the Mid-Domain Effect in Peak Fruiting Phenology

Our analysis reveals significant temporal overlap in the fruiting phenology of different species within constrained seasonal windows, resulting in a distinct peak in the middle of the phenological sequence. The observed “bell-shaped” distribution observed (Figure 2), characterized by a mid-year peak flanked by depressions toward both the early and late months, may arise from the aggregation of numerous small-scale fruiting events with similar phenological timing [16,24]. It is crucial to emphasize that the MDE model represents a neutral, geometric null expectation, relying solely on the random arrangement of phenological events within a bounded domain. These results indicate that the MDE model effectively explains a substantial portion of temporal variation in fruiting phenological diversity. Consequently, we recommend that future studies incorporate MDE null models to better disentangle the key drivers shaping seasonal patterns of phenological diversity.

Our findings align with previous research on the MDE in flowering phenology [15,16] but extend them in several critical dimensions. First, by analyzing a comprehensive dataset of 12,179 species, we demonstrate that the MDE is a pivotal mechanism not only in flowering but also in fruiting phenology. This underscores its broad relevance across reproductive phases. Most importantly, our study uncovers a distinct latitudinal distribution pattern of the MDE in fruiting phenology, different from that observed in flowering phenology. This divergence suggests that fruiting and flowering are influenced by distinct selective pressures, even within a shared geometric constraint. While the neutral MDE framework predicts the overall peak in temporal overlap, the contrasting latitudinal responses of flowering and fruiting likely arise because the former is primarily constrained by climatic cues that optimize pollination success, whereas the latter is more strongly shaped by post-pollination processes such as seed development rates and predation risk [22,23]. Overall, our work reinforces the MDE as a fundamental mechanism underlying phenological diversity patterns and deepens understanding of how geometric constraints specifically shape fruiting phenology.

The extremely low R^2^ in Hainan and the exceptionally high R^2^ in Xinjiang can be better understood when considering the interaction between domain length, floristic composition, and climate–null discrepancies. Hainan, which hosts the longest fruiting domain (12 months) and the highest year-round climate stability, contains a very large number of genera with highly asynchronous fruiting schedules. This combination weakens geometric constraints because an extended domain dilutes the overlap expected under a temporal mid-domain effect; even substantial variation in species’ fruiting durations produces only weak central aggregation. In contrast, Xinjiang exhibits one of the shortest effective fruiting domains (May–October), combined with a comparatively depauperate flora and strong climatic seasonality. Under these conditions, species’ phenophases are necessarily compressed into a narrow seasonal window, amplifying geometric overlap and yielding a near-perfect null-model fit. In addition, the climate–null mismatch differs markedly between the two regions: the weak seasonality of Hainan allows species to fruit opportunistically across many months, reducing alignment with any neutral geometric expectation, whereas the harsh thermal and hydric constraints in Xinjiang strongly synchronize reproductive timing, causing the empirical pattern to converge toward the geometric prediction. These contextual factors together explain why the MDE signal is minimal in Hainan but maximized in Xinjiang, and they highlight how domain size, floristic heterogeneity, and climatic forcing jointly modulate the strength of temporal geometric constraints. Importantly, causal directionality cannot be inferred from observational data, and the strong explanatory power of the neutral MDE model does not imply that adaptive processes are absent. Instead, it highlights that neutral geometric constraints alone can generate patterns resembling those produced by selection. Thus, any adaptive interpretation of latitudinal differences in phenophase duration must remain cautious, as neutral null models cannot identify the evolutionary drivers underlying the observed phenological structure.

A pivotal finding of this study is that the explanatory power of the MDE model peaks in mid-latitude regions. Notably, it does not increase monotonically with latitude. This non-monotonic pattern suggests that the effect of the neutral geometric constraint is modulated by adaptive life-history strategies that shape phenological trait distributions along the latitudinal gradient. This pattern is consistent with our theoretical framework. The domain size of fruiting (i.e., the bounded fruiting season) generally decreases with latitude, which would theoretically strengthen the MDE due to more stringent geometric constraints. However, the fruiting period of individual population also shortens with latitude. This reduction in phenological breadth likely represents an adaptive response to high-latitude environmental pressures (e.g., shorter growing seasons) and weakens the MDE signal, as geometric constraints exert less influence on species with very brief phenological durations [25]. Therefore, the explanatory power of the MDE is maximized in mid-latitude regions, where the interplay between domain size and species’ phenological breadths is optimal. This implies that mid-latitude regions represent a midpoint in the differentiation of fruiting strategies and may be particularly susceptible to future climate change impacts. The latitudinal variation in phenological breadth reflects adaptive strategies. Low-latitude species tend to adopt a “time for safety” strategy, indirectly extending population-level fruiting by increasing intraspecific variation in fruiting time (Figure 1). In contrast, high-latitude species tend to adopt a “quantity for safety” strategy, achieving predator saturation through concentrated, synchronous fruiting over a short period (Figure 1). Thus, the observed unimodal pattern arises from the interaction between the neutral geometric constraint (MDE) and adaptive strategies that shape phenological duration.

The consistent unimodal curves observed for both woody and herbaceous plants indicate that our theoretical framework possesses broad applicability across plant life forms. Consistent with Du et al. [16], our findings suggest that the classic spatial paradigm for community assembly does not fully apply to temporal niches. This distinction can be clarified by examining the different mechanisms driving spatial versus temporal niches. The filtering mechanism governing species distribution (spatial niche axis) likely reflects the evolutionary adaptation to climatic conditions. In contrast, the mechanism regulating reproductive timing along the temporal niche axis is more sensitive to short-term, interannual climate variability, suggesting fundamentally different underlying processes. From a temporal niche perspective, mid-latitude regions are largely constrained by the phenological window, a temporal manifestation of the mid-domain effect. Based on these findings, we speculate that fruiting strategies of mid-latitude plants, which require precise temporal matching, may face particular challenges under intensified climate change. This potential vulnerability arises because mid-latitude regions sit at the intersection of two opposing constraints: the fruiting domain becomes sufficiently narrow to impose strong geometric overlap, yet species’ phenophase durations have not contracted to the extent observed at high latitudes. In this intermediate regime, geometric overlap depends sensitively on the relative scaling between domain length and species-level phenological breadth. Climate-driven shifts in seasonal timing or domain boundaries—whether through changes in temperature, precipitation seasonality, or growing-season length—could therefore alter the balance that currently maximizes overlap. Testing this prediction could be a valuable goal for future studies that integrates direct fitness measurements with climate simulation models.

The differences in latitudinal patterns between fruiting and flowering phenology can be interpreted through our theoretical framework that moderates geometric expectations with climate-filtered life strategies. We infer that plants experience distinct natural selection pressures during flowering and fruiting phases, leading to the evolution of divergent adaptative strategies. At low latitudes, strong seed predation and reliance on specialized pollinators are hypothesized to favor highly synchronous flowering to match pollinator activity, but staggered fruit maturation to reduce per capita predation risk (Figure 1). At high latitudes, however, short growing seasons and predominantly generalist pollinators shift these pressures: asynchronous flowering maximizes the narrow window for pollination, whereas fruiting is more temporally concentrated, consistent with predator-satiation dynamics (Figure 1). Together, these opposing constraints help explain the divergent phenological strategies observed across the gradient. This theoretical framework draws support from established concepts in ecology, including predator satiation [22], pollinator competition [26], and economies of scale in reproduction [27]. Furthermore, the contrasting patterns between flowering and fruiting phenology suggest potential differences in underlying plant strategies. We interpret this as a conceptual moderation of the mid-domain effect, where climate-filtered life histories may interact with geometric constraints. It is critical to note that this integrative explanation remains an inferential framework rather than a demonstrated causal mechanism, and warrants future investigation.

### 3.2. The Mid-Domain Effect as a Driver of Fruiting Diversity

Our study systematically quantified the relative contributions of the MDE and climate variables to plant fruiting phenology. Our results showed that the MDE is the predominant factor shaping fruiting patterns in both herbaceous and woody plant communities. Specifically, the fruiting diversity in herbaceous communities shows significant correlations with the MDE and temperature, suggesting that climate warming could shift fruiting phenology. In contrast, fruiting diversity in woody plants is not only related to the MDE and temperature but also linked to precipitation. This indicates a generally stronger dependence of woody plants on water availability during the fruiting period, implying that changes in precipitation regimes—such as reduced frequency or extreme rainfall events—could significantly impact their reproductive processes [28,29]. This divergence likely stems from two key physiological mechanisms. Firstly, woody plants are typically perennial and maintain large biomass, requiring substantial photosynthetic activity during reproduction, which often creates a heightened dependence on water [30,31]. Secondly, woody plants rely on transpiration for thermoregulation and physiological balance [32,33,34]. Under sufficient sunlight, this results in greater water demand to maintain internal homeostasis. Consequently, water availability will directly affect physiological processes and reproductive investment in woody plants [35,36]. This ‘woody plant’ category inherently encompasses a diversity of water-use strategies (e.g., deep-rooted trees vs. drought-deciduous shrubs), and not all species exhibit uniformly high water demand. However, the physiological mechanisms described above—such as deeper root systems and greater carbon allocation to fruit development—provide a general framework that explains why, as an aggregate, woody plant fruiting phenology in our system exhibited a stronger response to precipitation than herbaceous species.

Our research further reveals that the MDE exerts a stronger influence on fruiting phenology than it does on flowering phenology [16]. This difference can be attributed to natural selection pressures outlined in our theoretical framework. While flowering phenology must align with pollinators that respond to short-term climatic cues—making it less predictable by purely geometric MDE models—fruiting phenology is shaped by strategies aimed at either avoiding seed predators or achieving predator satiation. The implementation of these predator-mediated mechanisms operates under stricter temporal constraints defined by the bounded season, thereby enhancing the explanatory power of the MDE model for fruiting events by changing phenophase duration. Although the MDE is often treated as a neutral null model, its strong explanatory power for fruiting phenology likely stems from adaptive processes such as predator satiation. This indicates that empirical patterns aligned with MDE may arise from both geometry and evolved fruiting strategies.

### 3.3. Limitations of the Study

The theoretical framework proposed in this study successfully predicted MDE patterns in fruiting phenology. However, this framework requires further empirical validation. Future research could involve controlled experiments in climate chambers or common garden settings, as well as analyses using higher-resolution phenological data. Quantifying key ecological variables, such as pollinator visitation frequency and seed predation rates, would provide direct evidence for shifts in plant “synchronization” or “dispersal” strategies.

While our analysis demonstrates the utility of floristic data for revealing broad-scale patterns, it is important to acknowledge the inherent limitations of this data source. Floristic compilations themselves may contain temporal biases, such as uneven sampling effort across seasons and the inherent challenge of precisely recording phenological events that span long periods. Consequently, representing fruiting phenology using median months remains an approximation. Although the prevalence of right-skewed curves implies that this method yields conservative estimates [37], and it remains a well-established approach in reproductive phenology research [38,39,40], we note that these potential biases are likely consistent across the latitudinal gradient and thus unlikely to generate the systematic unimodal pattern we discovered. Future studies using finer temporal-resolution data, such as from phenocams or herbarium records, will be invaluable for validating these patterns at a more precise, individual-level scale.

When evaluating the contribution of the MDE to actual phenological patterns, the delineation of boundaries needs to be particularly cautious, as the morphology of the MDE generated by simulations and the location of their peaks depend on the way boundaries are set. To further assess the robustness of the inferred latitudinal peak to boundary definition, we compared results obtained under the 5th–95th percentile and 10th–90th percentile thresholds. The unimodal pattern was preserved across all taxonomic groups, and the estimated peak latitudes remained numerically similar. For all species combined, the peak shifted from 39.6° N (5th–95th) to 36.2° N (10th–90th); for herbaceous species, the peak changed only slightly from 37.1° N to 37.8° N; and for woody species, it changed from 36.8° N to 35.7° N. Thus, the maximum shift observed across thresholds was 3.4°, and all peaks remained within a narrow mid-latitude band (approximately 35–40° N). These results indicate that the location of the latitudinal peak is highly stable with respect to reasonable variations in seasonal boundary definition.

Our use of a linear-domain representation follows approaches commonly adopted in previous temporal MDE studies [16] and is suited to datasets in which seasonal boundaries are asymmetric or weakly defined, as is the case for several tropical provinces in our analysis. Applying a circular model under such circumstances can introduce potential artefacts, because the December–January transition may not correspond to a biologically meaningful boundary in regions lacking clear seasonality. In our dataset, cross-year fruiting events appear infrequent after defining the 5th–95th percentile seasonal domain—only six provinces retain any records spanning December–January—so allowing wrap-around placement could artificially enlarge the effective domain and thereby increase the expected geometric overlap. Sensitivity analyses using alternative percentile thresholds (10th–90th percentiles), in which only one province showed evidence of cross-year overlap, produced similar MDE fits. This suggests that our main conclusions are robust to reasonable variations in boundary definition and to the treatment of potential wrap-around events.

## 4. Materials and Methods

### 4.1. Species Distribution and Phenology Data

This study focuses on analyzing the mid-domain effect at the provincial level. Species distribution data were obtained from the provincial floras. To align species distribution information across provincial Floras, we extracted a geographic centroid for each species within each province. We acknowledge that such centroids do not reflect the actual spatial extent or sampling coverage of species’ distributions; rather, they serve as a simplifying standardization procedure that allows each species to be consistently assigned to the provincial level. Because our analyses focus on temporal phenology and not on modelling fine-scale spatial distributions, using centroids provides a neutral and comparable representation across provinces without implying any biological accuracy regarding local habitat occupancy. We compiled fruit phenological data from 28 provincial floras. Then, we extracted three key pieces of information for each species from the floras: the earliest fruiting month, the latest fruiting month, and life form (woody or herbaceous). The fruiting period for each species was estimated as the median of its earliest and latest fruiting months. While this approach may introduce some biases, it is widely adopted in community-level and botanical studies where precise, continuous phenological records are unavailable [16,38,39,40,41,42,43]. Using the median fruiting month as the central phenological metric is methodologically justified for floristic sources where only the starting and ending months are available. Previous methodological comparisons have shown that, when phenological ranges are expressed as discrete month intervals, the median performs as a robust estimator of the phenophase centroid and is less sensitive to asymmetric or right-skewed distributions than simple midpoints [37,41]. Although alternatives such as length-weighted centroids can be used when continuous or high-resolution observations exist, these metrics converge closely with the median for the coarse-resolution, range-based records typical of provincial floras, with reported deviations generally under 1 month in broad-scale analyses. To minimize uncertainty, we applied strict filtering criteria and retained only species with explicitly documented month ranges (e.g., “May–July”), excluding entries with vague seasonal categories (e.g., “summer-autumn”). Based on well-established botanical criteria, we estimate that the proportion of ambiguous entries for each province does not exceed 5%. Collectively, these considerations support the use of the median as a reliable and conservative estimate of central fruiting timing for large-scale phenological synthesis. The final dataset included fruiting phenology records for 12,179 species, representing 2187 genera and 209 families (including 4638 woody and 7541 herbaceous species).

### 4.2. Climatic Data

Plant fruiting is known to be regulated by key environmental factors including precipitation, temperature, and photoperiod [7,8]. To investigate the influence of climate on monthly fruiting richness, we obtained meteorological data for the period 2015–2020 from the China Meteorological Data Service Centre (http://data.cma.cn). These data are in situ observations from ground-based weather stations across China. A single value for each climatic variable per province was obtained by extracting its value at the province’s geographic centroid. This approach provides a standardized provincial-level climatic baseline for inter-provincial comparison, rather than representing the microclimatic conditions that influence local phenology. The selected climatic variables were mean minimum monthly temperature (°C, Tmin), mean monthly precipitation (mm, MMP), and mean monthly sunshine duration (hours, Sunshine), which serve as proxies for thermal conditions, water availability, and photoperiod, respectively. Sunshine duration serves as a proxy for seasonal light availability, as it captures the actual duration of sunlight exposure, which is conceptually and practically linked to photoperiodic effects on plant phenology [44,45]. Although anomalies were not computed, our analysis relies on multi-year averages, a method that minimizes the effect of extreme inter-annual variability. This provides a robust climatological baseline and aligns with standard practices in biogeography [16,46].

### 4.3. Data Analyses

To test whether the mid-domain effect (MDE) explains the observed peak in fruiting periods, we employed a null model to simulate monthly fruiting species richness under geometric constraints [25]. The MDE test was conducted independently for each of the 28 provinces in China. Following the random placement model proposed by Colwell & Lees (2000) [25], we generated predicted monthly species richness patterns. The fruit period of each province was defined by the starting time, ending time, and duration. For instance, in Jilin Province, the fruiting domain was set from May to October based on the recorded ranges of all species. Within this bounded period, each species was randomly assigned a fruiting schedule according to its actual duration. The randomization process altered only the temporal placement (i.e., the start date or midpoint) of each species’ fruiting period. Crucially, the observed fruiting duration for each species was preserved exactly during the randomization process. The model first randomly selects the observed fruiting duration for each species and then randomly places it within the predefined temporal domain. This method thereby eliminates biases arising from discrepancies between theoretical and observed distributions. We applied a bootstrapping resampling approach (with replacement) to randomize the observed fruiting ranges, assigning a new random median value to each species based on its potential fruiting period midpoints [15]. Simulations were implemented using the “rangemodelR” package in R [47], with 5000 random iterations per province. The influence of MDE on monthly variation in fruiting species richness was evaluated by calculating the mean species richness per month and its 95th confidence interval across all simulations.

To avoid ambiguity associated with circular phenological data and prevent artificial overlap artifacts at the December–January calendar boundary, our null model represents the fruiting season as an open, linear interval rather than a cyclic (calendar-year) axis. Operationally, this means that the randomized placement of species’ fruiting periods does not allow “wrap-around” across December–January. This treatment follows directly from the structure of the empirical dataset: after defining the effective seasonal domain using the 5th–95th percentiles of cumulative fruiting records, the remaining observations contain only very sparse evidence of cross-year fruiting. Consequently, the truncated domain captures the period in which the overwhelming majority of fruiting activity is observed. Within this bounded interval, the observed duration of each species is preserved, and the randomization procedure restricts simulated placements to lie entirely within the empirically defined domain—i.e., it constrains the null model rather than making assumptions about the biological impossibility of cross-year fruiting. This approach clarifies how potential wrap-around events are handled and avoids introducing artefactual overlap that would arise from imposing a circular model on data with asymmetrically bounded seasonal activity.

We used ordinary least squares linear regression to assess the relationship between the observed number of fruiting species and the MDE model predictions. The goodness-of-fit for each province was quantified using the coefficient of determination (R^2^) and significance level (*p*-value). Separate regression analyses were conducted for all species, herbaceous plants, and woody plants to examine the latitudinal patterns in MDE temporal geometric fit.

Given that the shape and midpoint of the MDE are highly dependent on predefined boundaries, special consideration was given to provinces with potentially year-round fruiting activity, such as the southernmost provinces. To improve model applicability in regions with less distinct phenological boundaries, we adopted a more stringent criterion by defining the boundaries at the 5th and 95th percentiles of cumulative fruiting records [16]. We adopted the 5th and 95th percentiles as temporal boundaries because this percentile-based approach has been shown in simulation studies to minimize the influence of outlier records and reduce artificial inflation of the phenological domain in regions with year-round phenological activity [16]. To verify that our results were not an artifact of this specific threshold, we conducted a sensitivity analysis using alternative boundary definitions (10th/90th percentiles). The resulting MDE fits and latitudinal patterns were nearly identical, demonstrating that our key conclusions are robust to reasonable variations in domain definition (see Supplementary Table S1; Figures S1 and S2).

To quantify the relative contributions of the MDE and climate variables to observed fruit patterns, we constructed a linear mixed effects model with the actual monthly number of species as the response variable. Fixed effects included MDE-predicted monthly fruiting species count, mean minimum monthly temperature (°C, Tmin), total monthly precipitation (mm, MMP), and total monthly sunshine duration (h, Sunshine). Month was included as a random intercept to account for temporal pseudoreplication. The continuous predictor variables were included in their original scales to facilitate the interpretation of regression coefficients in their natural units. To compare effect sizes across predictors measured in different units, we reported semi-standardized coefficients (βss), calculated as the raw coefficient multiplied by the ratio of the predictor’s standard deviation to the response variable’s standard deviation. Models were fitted using the “lmer” function from the R package “lme4”. Variance inflation factor (VIF) tests confirmed the absence of significant multicollinearity among predictor variables (all VIF < 3, Table S2). We adopted this more conservative threshold (compared to the common benchmark of VIF < 5 or 10) to ensure greater robustness of our models against multicollinearity [48]. The independent contributions of each fixed factor were evaluated using the “glmm.hp” package in R, which is specifically designed for variance partitioning in mixed models [49,50]. All predictors were standardized. The intraclass correlation coefficient (ICC) of the random effect (Month) are reported in the Supplementary Material (Table S2).

To investigate the latitudinal gradient pattern of the MDE during the fruiting period, we examined the relationship between the independent explanatory power of the MDE and latitude using both linear and nonlinear regression models. The optimal model was selected by comparing the Akaike Information Criterion (AIC) to identify the most representative latitudinal pattern. These regression analyses were performed separately for all species, herbaceous species, and woody species to determine whether the latitudinal patterns of the MDE were consistent across different plant life forms. All analyses were conducted in R version 4.4.1 [51].

## 5. Conclusions

This study demonstrates that the mid-domain effect is a key mechanism shaping fruiting phenology across China. Using data from over 12,000 species, we found that the observed fruiting richness strongly aligns with the predictions of the MDE null model, thus confirming a substantial influence of geometric constraints on reproductive timing, while climate factors and adaptation likely moderate the realized structure. The explanatory power of the MDE follows a unimodal latitudinal pattern, peaking at mid-latitudes (36.8–39.6° N), consistent with our theoretical framework. This contrasts with the linear trend reported for flowering phenology, indicating that fruiting and flowering respond to distinct selective pressures. The MDE contributed more strongly than climatic factors, though woody plants showed additional sensitivity to precipitation. Overall, this study extends the application of the MDE from spatial and flowering contexts to fruiting phenology, offering a unifying geometric mechanism for understanding temporal biodiversity patterns. The observed unimodal latitudinal pattern provides a new perspective for informing climate change models that track reproductive timing, especially for predicting shifts in mid-latitude regions where geometric constraints and environmental factors interact most strongly. Future research combining fine-scale phenological observations with experimental and modeling approaches will be crucial for verifying the adaptive and ecological bases of these patterns. These insights offer practical implications for ecological monitoring and conservation planning. In particular, the identification of mid-latitude regions as zones of heightened phenological sensitivity suggests that monitoring networks should prioritize finer temporal resolution in these areas, and conservation strategies should incorporate phenology-based indicators to detect early signs of climate-driven disruption in fruiting dynamics.

## Figures and Tables

**Table 1:** Summary of the linear regression models of the relationship between the observed number of species fruiting and the mid-domain effect model-predicted number of species fruiting, based on the 5th and 95th percentiles of cumulative fruiting records. F is the F-statistic testing the overall model significance by comparing explained and unexplained variance. Bold indicates significance (*p* < 0.05).

Province	Latitude	All Species				Herbaceous Species				Woody Species			
		F	R^2^	*p*	Period	F	R^2^	*p*	Period	F	R^2^	*p*	Period
Hainan	19.222	1.2	0.107	0.299	1–12	3.3	0.250	0.098	1–12	0.3	0.033	0.573	1–12
Guangxi	23.015	1.6	0.135	0.239	1–12	3.6	0.266	0.086	1–12	0.7	0.069	0.408	1–12
Guangdong	23.277	1.8	0.156	0.204	1–12	4.3	0.299	0.066	1–12	0.8	0.077	0.382	1–12
Taiwan	23.657	2.9	0.224	0.120	1–12	5.7	**0.361**	0.039	1–12	1.0	0.090	0.345	1–12
Yunnan	24.141	2.7	0.216	0.128	1–12	10.5	**0.566**	0.012	3–12	1.6	0.141	0.230	1–12
Fujian	26.004	3.3	0.249	0.099	1–12	5.5	**0.355**	0.041	1–12	1.7	0.144	0.224	1–12
Guizhou	26.668	16.2	**0.699**	0.005	4–12	29.4	**0.807**	<0.001	4–12	9.8	**0.584**	0.016	4–12
Jiangxi	27.735	19.4	**0.735**	0.003	4–12	51.4	**0.880**	<0.001	4–12	7.9	**0.532**	0.026	4–12
Hunan	28.016	20.0	**0.741**	0.003	4–12	49.6	**0.876**	<0.001	4–12	10.0	**0.589**	0.016	4–12
Zhejiang	29.105	19.3	**0.734**	0.003	4–12	14.3	**0.704**	0.009	4–11	11.0	**0.647**	0.016	4–12
Sichuan	30.277	14.9	**0.680**	0.006	4–12	12.4	**0.674**	0.012	4–11	8.5	**0.549**	0.022	4–12
Xizang	31.101	14.3	**0.705**	0.009	5–12	32.9	**0.868**	0.002	5–11	8.2	**0.538**	0.024	4–12
Anhui	32.014	23.8	**0.773**	0.002	4–12	45.9	**0.868**	<0.001	4–12	8.0	**0.533**	0.025	4–12
Hubei	32.014	16.4	**0.701**	0.005	4–12	11.2	**0.652**	0.015	4–11	11.4	**0.620**	0.012	4–12
Jiangsu	32.472	30.6	**0.814**	<0.001	4–12	51.2	**0.880**	<0.001	4–12	9.9	**0.586**	0.016	4–12
Henan	33.8	10.6	**0.639**	0.017	4–11	18.7	**0.757**	0.005	4–11	9.5	**0.656**	0.027	5–11
Shanxi	34.115	15.8	**0.725**	0.007	4–11	23.0	**0.793**	0.003	4–11	10.9	**0.686**	0.021	5–11
Qinghai	35.723	26.6	**0.869**	0.007	5–10	35.3	**0.898**	0.004	5–10	8.7	**0.686**	0.042	5–10
Gansu	35.949	33.4	**0.870**	0.002	5–11	41.7	**0.893**	0.001	5–11	11.1	**0.690**	0.021	5–11
Shandong	36.178	16.5	**0.733**	0.007	4–11	30.3	**0.835**	0.002	4–11	3.7	0.380	0.103	4–11
Ningxia	37.366	15.4	**0.794**	0.017	5–10	25.6	**0.865**	0.007	5–10	5.0	0.556	0.089	5–10
Shaanxi	37.699	22.7	**0.820**	0.005	5–11	12.0	**0.666**	0.013	4–11	10.8	**0.683**	0.022	5–11
Hebei	38.222	16.0	**0.727**	0.007	4–11	19.7	**0.766**	0.004	4–11	12.5	**0.714**	0.017	5–11
Inner Mongolia	41.386	19.0	**0.826**	0.012	5–10	23.2	**0.853**	0.009	5–10	7.0	0.637	0.057	5–10
Liaoning	41.474	6.3	0.613	0.066	5–10	3.8	0.429	0.110	4–10	1.4	0.256	0.306	5–10
Xinjiang	42.002	87.0	**0.956**	<0.001	5–10	111.5	**0.965**	<0.001	5–10	15.6	**0.796**	0.017	5–10
Jilin	43.501	9.3	**0.699**	0.038	5–10	11.4	**0.739**	0.028	5–10	3.6	0.472	0.132	5–10
Heilongjiang	46.77	39.8	**0.888**	0.001	5–11	20.9	**0.777**	0.004	5–11	3.3	0.453	0.143	5–10

**Table 2:** Parameters from linear mixed-effects models (lmer) modeling the number of species fruiting as a function of mid-domain effect, mean minimum monthly temperature (°C, Tmin), mean monthly precipitation (mm, MMP), and mean monthly sunshine duration (h, Sunshine), with a random intercept for month, based on the 5th and 95th percentiles of cumulative fruiting records. The ‘I.perc’ column shows the individual contribution (%) as calculated by the glmm.hp function.

	All Species			Herbaceous Species			Woody Species		
	t	*p*	I.perc (%)	t	*p*	I.perc (%)	t	*p*	I.perc (%)
mid-domain effect	21.4	<0.001	89.18	26.3	<0.001	92.96	23.0	<0.001	72.85
Tmin	3.0	0.002	6.43	3.6	<0.001	4.41	5.0	<0.001	15.50
MMP	−1.2	0.234	2.73	−0.8	0.423	1.20	−2.5	0.012	6.71
sunshine	−0.9	0.350	1.67	−1.9	0.059	1.44	0.24	0.811	4.93

## Data Availability

The original contributions presented in the study are included in the article, further inquiries can be directed to the corresponding author.

## Supplementary Materials

The following supporting information can be downloaded at: https://www.mdpi.com/article/10.3390/plants14233701/s1, Figure S1: The relationship between the variance explained by the mid-domain effect models and latitude for all species (a), woody species (b) and herbaceous species (c), based on the 10th and 90th percentiles of cumulative fruiting records. Each circle represents a single province. The solid red curve is the fitted line from the nonlinear model. The gray dashed line is the fitted line from the linear model. The red vertical dashed line represents the peak of the quadratic regression curve. ∆AIC is the difference in AIC between two models, and a ∆AIC greater than zero indicates better fitting of the nonlinear model; Figure S2: Observed and null model-predicted fruiting diversity for 28 provinces, based on dates for which 10th and 90th of cumulative fruiting records occurred. The fitted values (solid lines) and the 95% confidence interval predicted by the mid-domain models (gray bands) are shown in the plots. Each panel represents one province, labeled with the province name. The R2 and P-values reflect the relationship between the observed data and the predicted values from the mid-domain model; Table S1: Summary of the linear regression models of the relationship between the observed number of species fruiting; Table S2: Model diagnostics for the mixed-effects models examining the effects of predictors on fruiting species richness.

## Abbreviations

The following abbreviations are used in this manuscript:

MDE	Mid-domain effect
