# Supplementary material for: The Mid-Domain Effect Shapes a Unimodal Latitudinal Pattern in Fruiting Phenology
Source: Plants (Basel). 2025 Dec 4;14(23):3701. doi: 10.3390/plants14233701 (PMC12694029; doi:10.3390/plants14233701)
Supplement: Supplementary file 1 [file plants-14-03701-s001.zip › plants-3980160-supplementary.pdf]

Table S1. Summary of the linear regression models of the relationship between the observed number of species fruiting and the mid-domain effect model-predicted number of species fruiting, based on the 10th and 90th percentiles of cumulative fruiting records. F is the F-statistic testing the overall model significance by comparing explained and unexplained variance. Bold indicates significance ($P < 0.05$).

		All species				Herbaceous species				Woody species			
Province	Latitude	F	R^2	*P*	Period	F	R^2	*P*	Period	F	R^2	*P*	Period
Hainan	19.222	1.2	0.107	0.299	1-12	3.3	0.250	0.098	1-12	0.3	0.033	0.573	1-12
Guangxi	23.015	9.1	**0.566**	0.019	4-12	6.0	0.037	0.400	2-12	0.7	0.069	0.408	1-12
Guangdong	23.277	11.5	**0.622**	0.012	4-12	29.6	**0.809**	<0.001	4-12	0.8	0.077	0.382	1-12
Taiwan	23.657	4.9	0.352	0.054	2-12	69.3	**0.908**	<0.001	4-12	1.0	0.090	0.345	1-12
Yunnan	24.141	36.5	**0.859**	<0.001	5-12	40.4	**0.871**	<0.001	5-12	18.0	**0.750**	0.005	5-12
Fujian	26.004	21.0	**0.750**	0.003	4-12	52.5	**0.882**	<0.001	4-12	8.4	**0.547**	0.023	4-12
Guizhou	26.668	35.1	**0.854**	0.001`	5-12	5.9	0.500	0.051	4-11	22.2	**0.787**	0.003	5-12
Jiangxi	27.735	40.1	**0.870**	<0.001	5-12	7.7	**0.561**	0.032	4-11	15.8	**0.724**	0.007	5-12
Hunan	28.016	46.0	**0.885**	<0.001	5-12	8.7	**0.593**	0.025	4-11	23.5	**0.797**	0.003	5-12
Zhejiang	29.105	9.0	**0.643**	0.030	5-11	14.3	**0.704**	0.009	4-11	6.7	0.627	0.061	6-11
Sichuan	30.277	21.4	**0.811**	0.006	5-11	44.8	**0.900**	0.001	5-11	16.1	**0.728**	0.007	5-12
Xizang	31.101	24.9	**0.833**	0.004	5-11	32.9	**0.868**	0.002	5-11	9.4	**0.654**	0.028	5-11
Anhui	32.014	7.7	**0.561**	0.032	4-11	19.6	**0.766**	0.004	4-11	5.0	0.498	0.076	5-11
Hubei	32.014	18.6	**0.788**	0.008	5-11	34.8	**0.874**	0.002	5-11	10.3	**0.673**	0.024	5-11
Jiangsu	32.472	10.0	**0.625**	0.019	4-11	19.4	**0.763**	0.005	4-11	5.7	0.533	0.062	5-11
Henan	33.8	31.5	**0.863**	0.002	5-11	18.7	**0.757**	0.005	4-11	9.5	**0.656**	0.027	5-11
Shanxi	34.115	6.1	0.604	0.069	5-10	11.0	**0.734**	0.029	5-10	10.9	**0.686**	0.021	5-11
Qinghai	35.723	43.8	**0.936**	0.007	6-10	31.2	**0.912**	0.011	6-10	51.2	**0.945**	0.006	6-10
Gansu	35.949	5.6	0.584	0.077	5-10	10.7	**0.728**	0.031	5-10	33.8	**0.894**	0.004	6-11

Shandong	36.178	51.2	**0.911**	<0.001	5-11	30.3	**0.835**	0.002	4-11	3.7	0.380	0.103	4-11
Ningxia	37.366	15.4	**0.794**	0.017	5-10	25.6	**0.865**	0.007	5-10	5.0	0.556	0.089	5-10
Shaanxi	37.699	22.7	**0.820**	0.005	5-11	35.6	**0.877**	0.002	5-11	10.8	**0.683**	0.022	5-11
Hebei	38.222	8.0	**0.667**	0.047	5-10	12.6	**0.759**	0.024	5-10	12.5	**0.714**	0.017	5-11
Inner Mongolia	41.386	19.0	**0.826**	0.012	5-10	23.2	**0.853**	0.009	5-10	7.0	0.637	0.057	5-10
Liaoning	41.474	6.3	0.613	0.066	5-10	9.9	**0.713**	0.035	5-10	1.4	0.256	0.306	5-10
Xinjiang	42.002	87.0	**0.956**	<0.001	5-10	111.5	**0.965**	<0.001	5-10	15.6	**0.796**	0.017	5-10
Jilin	43.501	9.3	**0.699**	0.038	5-10	11.4	**0.739**	0.028	5-10	3.6	0.472	0.132	5-10
Heilongjiang	46.77	13.7	**0.774**	0.021	5-10	18.2	**0.820**	0.013	5-10	3.3	0.453	0.143	5-10

Table S2. Model diagnostics for the mixed-effects models examining the effects of predictors on fruiting species richness. The table presents the Variance Inflation Factor (VIF) for each fixed effect, semi-standardized coefficients(β ss) , and the Intraclass Correlation Coefficient (ICC) for the random effect (Month) across all species, herbaceous species, and woody species.

	All species			Herbaceous species			Woody species		
	VIF	βss	ICC	VIF	βss	ICC	VIF	βss	ICC
mid-domain effect	1.09	0.72		1.10	0.80		1.34	0.73	
Tmin	1.61	0.26		1.67	0.26		1.66	0.26	
MMP	2.21	-0.07		2.31	-0.04		2.30	-0.17	
sunshine	1.72	0.01		1.82	-0.01		1.74	-0.01	
Month			0.558			0.635			0.666

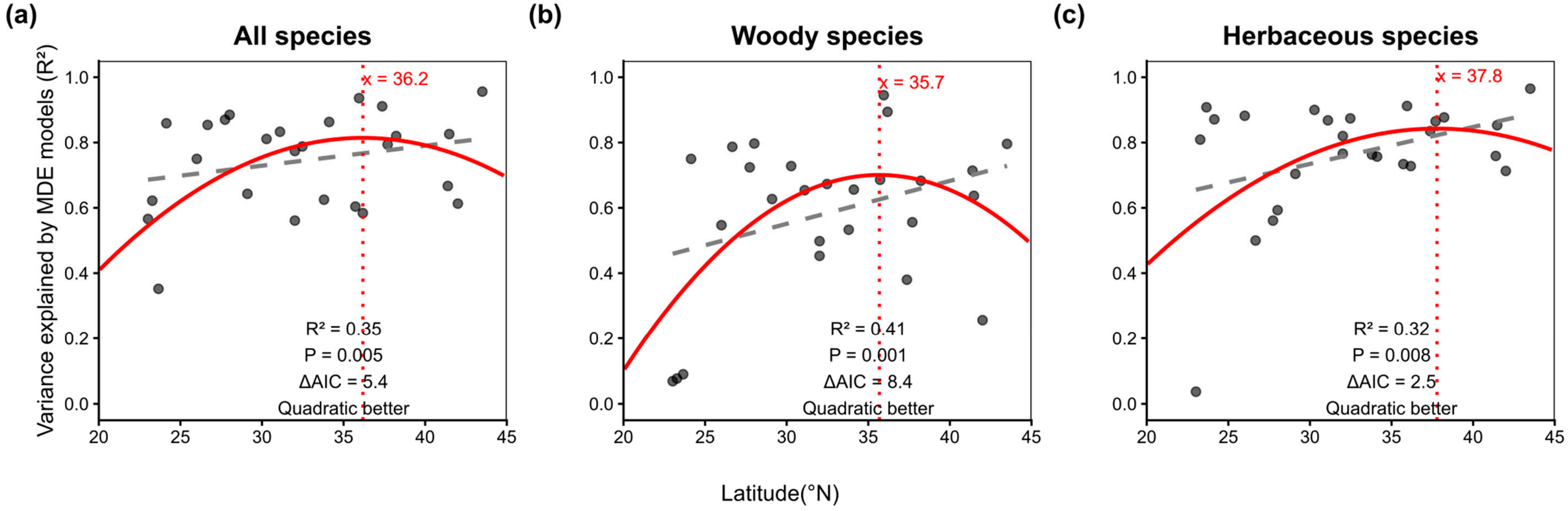

Figure S1. The relationship between the variance explained by the mid-domain effect models and latitude for all species (a), woody species (b) and herbaceous species (c), based on the 10th and 90th percentiles of cumulative fruiting records. Each circle represents a single province. The solid red curve is the fitted line from the nonlinear model. The gray dashed line is the fitted line from the linear model. The red vertical dashed line represents the peak of the quadratic regression curve.

ΔAIC is the difference in AIC between two models, and a ΔAIC greater than zero indicates better fitting of the nonlinear model.

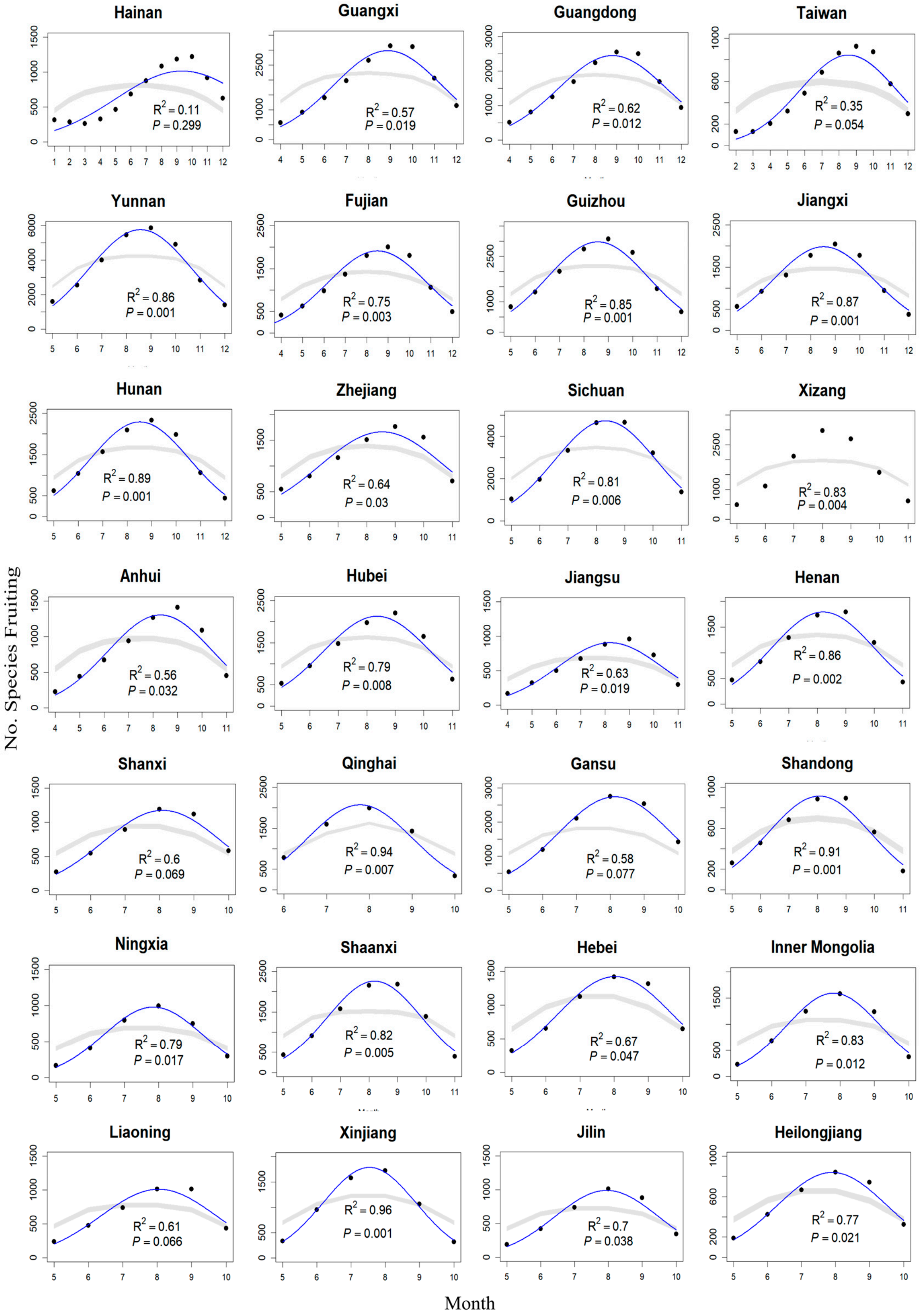

Figure S2. Observed and null model-predicted fruiting diversity for 28 provinces, based on dates for which 10th and 90th of cumulative fruiting records occurred. The fitted values (solid lines) and the 95% confidence interval predicted by the mid-domain models (gray bands) are shown in the plots. Each panel represents one province, labeled with the province name. The R^2 and P-values reflect the relationship between the observed data and the predicted values from the mid-domain model.
